# Supplementary figures and images for: Characteristics of transferrin saturation and anemia-related biomarkers in patients with uterine adenomyosis
Source: PLoS One. 2026 Mar 17;21(3):e0344781. doi: 10.1371/journal.pone.0344781 (PMC12994800; doi:10.1371/journal.pone.0344781)

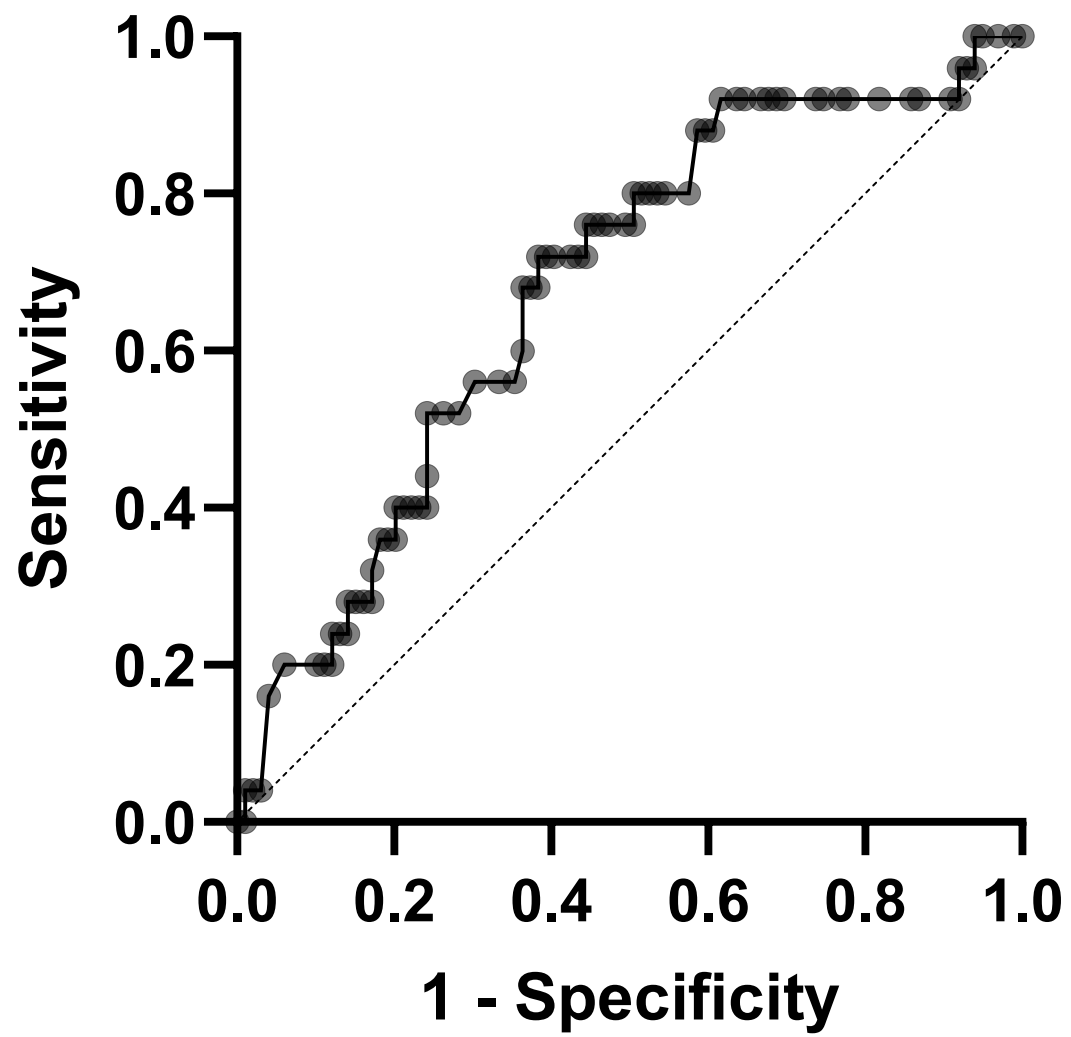

Supplement: S1 Fig — ROC curve of the multivariable logistic regression model for TSAT-only classification (TSAT <20% with ferritin ≥20 ng/mL) among patients with ferritin measured at the first visit (n = 124). Predictors included diagnosis group (adenomyosis and myoma, with no uterine structural abnormality as the reference), age (years), and hypermenorrhea. The area under the ROC curve (AUC) was 0.678 (95% CI 0.563–0.793). (PDF) [file pone.0344781.s001.pdf]
