# Supplementary material for: Characteristics of transferrin saturation and anemia-related biomarkers in patients with uterine adenomyosis
Source: PLoS One. 2026 Mar 17;21(3):e0344781. doi: 10.1371/journal.pone.0344781 (PMC12994800; doi:10.1371/journal.pone.0344781)
Supplement: Attachment — Submitted filename: Major_revision_20250916_M2.docx [file pone.0344781.s004.docx]

Additional Editor Comments:

#1

First, align the title, the aim stated in the Abstract, and the aim in the Introduction. If no intervention was analyzed, remove references to “therapeutic iron repletion.”

Response:

Thank you for pointing this out. We removed references to “therapeutic iron repletion.”

#2

Throughout, use frequency rather than prevalence.

Response:

We agree with the reviewer and have replaced “prevalence” with “frequency” throughout the manuscript, including headings and figure text.

#3

Define anemia and iron deficiency explicitly—distinguishing absolute from functional iron deficiency—and state clearly how ferritin and TSAT were used (individually and in combination).

Response:

We appreciate the suggestion. We added a Definitions subsection specifying the following: anemia = Hb <11 g/dL; absolute iron deficiency = ferritin <20 ng/mL; functional iron deficiency = TSAT <20%, with ferritin ≥20 ng/mL; overall iron deficiency = ferritin <20 ng/mL and/or TSAT <20%.

#4

Please revise the passages that might imply ferritin/TSAT diagnose anemia; anemia should be defined by hemoglobin, whereas ferritin/TSAT define iron deficiency.

Response:
We completely agree with your comment and reworded the Abstract, Results, and Discussion to avoid suggesting that ferritin/TSAT levels can be used to diagnose anemia. We have stated explicitly that anemia was defined using Hb only, whereas iron deficiency was defined using ferritin and/or TSAT.

#5

Second, clarify study design and cohort construction. Specify how the study population was selected and whether TSAT, ferritin, serum iron, and TIBC were ordered routinely or based on presenting complaints.

Response:

We appreciate the editor’s suggestion. We clarified that the study is a retrospective observational study of consecutive first-visit gynecology outpatients (Apr 2019–Jan 2025). CBC is routine at the first visit, whereas iron studies (serum iron, TIBC, ferritin)—from which TSAT is calculated—were ordered at the attending physician’s discretion based on clinical context. These clarifications were made by revising Study design and population.

#6

Reconcile the retrospective timeframe (2019–2025) with IRB approval and consent procedures.

Response:

We appreciate the suggestion. The study was conducted under our institution’s broad written consent program. The IRB approved the retrospective use of records from April 2019 to January 2025. Participants had already provided broad written consent permitting the use of de-identified clinical data for research.

#7

Define the target population and any clinician-applied exclusion criteria, explaining how selection bias was minimized.

Response:

Thank you for this helpful comment. We specified the target population as non-menopausal women aged 30–55 years who underwent first-visit blood testing. We listed prespecified exclusions. To minimize selection bias, we used consecutive sampling, applied a uniform first-visit laboratory policy, and limited exclusions to the above a priori criteria. These changes were made by revising Materials and Methods → Study design and population.

#8

Please also explain why missing ferritin disproportionately excluded patients in the myoma group compared with adenomyosis and controls.

Response:

Thank you for pointing this out. In Discussion (Limitations), we added that iron studies were not protocolized and were ordered at clinician discretion.

#9

Third, address clinical interpretation. Analyze whether symptom severity correlates with TSAT within adenomyosis and myoma (e.g., PBAC/menorrhagia and dysmenorrhea scores or appropriate binary proxies) and report effect sizes.

Response:

We appreciate the suggestion. Owing to the retrospective design, validated severity instruments (PBAC, dysmenorrhea VAS) were not systematically collected; only binary proxies (self-reported heavy menstrual bleeding and fatigue) were available. We therefore stratified by heavy menstrual bleeding (Fig. 4), where the Hb–TSAT correlation was absent in adenomyosis, supporting functional iron deficiency despite preserved Hb. We acknowledge this limitation and plan a prospective study collecting standardized severity measures to quantify severity–TSAT associations.

#10

Expand the Discussion on iron deficiency without anemia, and briefly comment on renal and cardiovascular consequences of iron deficiency and their relevance to interpreting TSAT in inflammatory states.

Response:

Thank you for this helpful comment. We expanded the final paragraphs of Introduction and Discussion to summarize iron deficiency without anemia and briefly noted the renal/cardiovascular effects of iron deficiency, emphasizing their relevance to interpreting TSAT under inflammatory conditions.

#11

Strengthen the mechanistic link among adenomyosis-related inflammation, hepcidin, and functional iron deficiency.

Response：

We appreciate the suggestion. We strengthened the mechanistic relationship in Introduction, outlining how adenomyosis-related inflammation can increase hepcidin, leading to functional iron deficiency (i.e., low TSAT levels, despite preserved ferritin levels).

#12

Moderate language that overstates a “lack of correlation.”

Response：

Thank you for this helpful comment. We have moderated the wording and revised the title to avoid implying an absolute lack of correlation.

Review Comments to the Author

Reviewer #1:

#13

Kindly use word Frequency instead of Prevalence.

Response:

We appreciate the suggestion. We agree with the reviewer and have replaced “prevalence” with “frequency” throughout the manuscript, including headings and figure text.

#14

Should also comment on kidney and cardiovascular dearragements that occur in iron deficiency. Should deliberate more in discussion regarding iron deficiency without anemia, as this is much prevalent.

Response:

Thank you for this helpful comment. We expanded the Introduction and Discussion sections to summarize iron deficiency without anemia and briefly noted the effects of iron deficiency on the renal/cardiovascular system, emphasizing their relevance to interpreting TSAT under inflammatory conditions.

Reviewer #2: This is a well thought out research. However, there are some areas that will need clarification, reconstruction and adjustment in order to present a logical conclusion some of which are highlighted below as questions, observations and suggestions.

#15

There is disconnection between the title, the aim as written in the abstract (line 27-28) and the study aim written in the introduction section (line 94-96).

Response:

We appreciate the reviewer’s point. We have harmonized the study aim across the title, Abstract, and Introduction. In Abstract, we removed treatment-leaning language and clarified the diagnostic focus.

#16

How was study population selected and what role did their presenting complain play in the selection?

Response:

We appreciate the opportunity to clarify this point. We clarified that this is a retrospective observational study of consecutive first-visit gynecology outpatients (Apr 2019–Jan 2025). CBC is routine at the first visit, whereas iron studies (serum iron, TIBC, ferritin)—from which TSAT is calculated—were ordered at the attending physician’s discretion based on clinical context. These clarifications were made by revising Study design and population.

#17

What is therapeutic iron repletion?

Response:

Thank you for pointing this out. We removed any reference to “therapeutic iron repletion” in the manuscript.

#18

How is functional iron deficiency diagnosed especially in this study population?

Response:

We appreciate the suggestion. We added a Definitions subsection specifying the following: anemia = Hb <11 g/dL; absolute iron deficiency = ferritin <20 ng/mL; functional iron deficiency = TSAT <20%, with ferritin ≥20 ng/mL; overall iron deficiency = ferritin <20 ng/mL and/or TSAT <20%.

#19

If this was a retrospective review, how was informed consent gotten from patients prior to participation in the study?

Response:

We appreciate the suggestion. The study was conducted under our institution’s broad written consent program. The IRB approved the retrospective use of records from April 2019 to January 2025. Participants had already provided broad written consent permitting the use of de-identified clinical data for research.

#20

What exactly is the study design used? it is noted also that ethical approval was gotten on 31/10/2023 but the study was a retrospective review from 01/04/2019 to 31/01/2025.

Response:

We are grateful for this suggestion. We have stated that the study is a retrospective observational analysis of consecutive first-visit gynecology outpatients. Regarding oversight and consent, we clarified that IRB approval (31 Oct 2023) was obtained under our institution’s broad written consent framework for secondary use of clinical data, which authorized the retrospective review of records from April 2019 to January 2025.

#21

The study design, participants recruitment is ambiguous. it needs to be better written.

Response:

We appreciate the reviewer’s point. We revised Study design and population to clarify the patient population (non-menopausal women aged 30–55 years with first-visit blood testing) included in the study. These clarifications were made by revising Study design and population.

#22

Who are the target population for this study?

Response:

Thank you for this helpful comment. We revised Study design and population to explicitly state the target population: non-menopausal women aged 30–55 years who presented for an initial gynecology visit and underwent first-visit blood testing.

#23

What factors were considered by the attending obstetrician-gynecologist to deem a patient unfit for participating in the study? How will that not introducing selection bias?

Response:

Thank you for highlighting this point. We agree that clinician-discretionary exclusion can introduce selection bias. In our study, no exclusions were made at clinicians’ discretion; only the prespecified, non-discretionary criteria outlined in Study design and population. We have removed expressions that could suggest clinician-discretionary exclusion. Consecutive enrollment was undertaken to minimize selection bias.

#24

Is it a routine practice to test for TSAT and serum ferritin levels? Was serum iron also assayed for

Response:

We appreciate the reviewer’s question. A complete blood count is routinely performed on the first visit. Iron studies (serum iron, TIBC, and ferritin, used to calculate TSAT) are ordered at the attending physician’s discretion according to clinical context. As this information is already specified in Study Design and Population, we did not make further textual changes.

#25

There were more patients excluded in the myoma group (49%) compared with Adenomyosis group (31%) and those without structural anomalies (20%) because of lack of ferritin assay. could you provide some explanation to this?

Response:

Thank you for pointing this out. We added to Discussion (Limitations) that iron studies were not protocolized and were ordered at the clinician’s discretion.

＃26

Table 1 included all the initial patients despite that some were excluded from the study because of lack of ferritin assay.

Response:

We appreciate the opportunity to clarify. Table 1 is now explicitly defined as the correlation cohort (patients with Hb and TSAT levels available at the first visit). In Results, we stated that two prespecified analysis populations were used: a correlation cohort (Hb and TSAT levels available; summarized in Table 1) and a ferritin analytic cohort (first-visit ferritin available) for iron-deficiency frequencies.

#27

Line 157 -163 needs to be written to ensure clarity. How is iron deficiency diagnosed using ferritin and TSAT combined as opposed to using ferritin or TSAT alone.

Response
Thank you for this helpful comment. We agree and rewrote the second sentence of the paragraph in Results titled “Frequency of anemia and iron deficiency in the groups differed according to different markers” to specify the three marker-based criteria and separate anemia.

＃28

What was the justification for the log transformation of TSAT? Did you consider a sensitivity analysis without the log transformation? Assuming the reason for the transformation was the non-normality of the data, did you consider doing a different form of correlation?

Response:

We appreciate the reviewer’s thoughtful suggestion. We clarified in Statistical analysis that TSAT was right-skewed; therefore, we log-transformed TSAT to better satisfy Pearson correlation assumptions. As a sensitivity analysis, Spearman’s rank correlation was performed on untransformed TSAT. We reported in Results that the qualitative group pattern was consistent in Spearman (adenomyosis showing the weakest Hb–TSAT association).

＃29

The pathophysiology of adenomyosis includes some chronic inflammatory process. what is the effect of that on the TSAT level as compared to the other groups?

Response:

Thank you for this helpful question. We strengthened the mechanistic context in Introduction, discussion how adenomyosis-related inflammation can increase hepcidin, leading to functional iron deficiency (i.e., low TSAT despite preserved/normal-to-elevated ferritin). We show that adding TSAT nearly doubles iron-deficiency detection in adenomyosis (31.0-65.5%)１but changes little in the myoma and non-structural control groups. Consistently, the Hb-TSAT correlation is weak in adenomyosis (r=0.21; with hypermenorrhea r=0.05) and strong in myoma (r=0.55) and controls (r=0.69). These comparisons are now clearly presented in the Results (Fig.2-4) and emphasized in the Discussion.

#30

What explanation can you give as to why the findings in this study was different from previous studies? (line 199-201)

Response:

Thank you for the thoughtful suggestion. We clarified this point in Discussion. Immediately after the sentence on mechanistic relationship, we added a sentence explaining the methodological differences, namely, the use of TSAT in addition to ferritin and the explicit evaluation of Hb–TSAT coupling.

＃31

Also since all the patients did not present with hypermenorrhea, were there other factors considered (as cofounders) that could have been responsible for the iron deficiency or anemia?

Response:

We appreciate the point. In this retrospective dataset, standardized measures for potential confounders beyond hypermenorrhea were not systematically available.

＃32

Line 209-214, This should be rewritten so as not to give the impression of comparing prevalence of anemia diagnosed by Hb concentration and ferritin. and since it is common knowledge that there is a subtype of Iron deficiency that do not present with anemia, the Hb levels cannot be used to diagnose iron deficiency and likewise ferritin and TSAT should not be used to diagnose anemia.

Response:

We agree with the reviewer’s comment. We rewrote the second paragraph of Discussion to clarify that anemia was defined using Hb only, whereas iron deficiency was defined using ferritin and/or TSAT, and we reported these as separate frequencies. We deleted expressions that could suggest direct comparison of “anemia diagnosed by ferritin” and instead described marker-based iron-deficiency frequencies alongside Hb-defined anemia.
