# Supplementary material for: Characteristics of transferrin saturation and anemia-related biomarkers in patients with uterine adenomyosis
Source: PLoS One. 2026 Mar 17;21(3):e0344781. doi: 10.1371/journal.pone.0344781 (PMC12994800; doi:10.1371/journal.pone.0344781)
Supplement: Attachment — Submitted filename: Reviewer Plos.docx [file pone.0344781.s003.docx]

Tittle page

- Authors are advised to review the journal’s author guidelines and follow a classic structure when submitting manuscripts.

Abstract

- A structured abstract is recommended.
- Establish cut-off points for anemia and iron deficiency for Hb, Ferritin, TSAT.
- The abstract refers to a strong correlation; however, the corresponding quantitative values and p-values are not reported.

Introduction

- Lines 76 to 78 contain references that are not up to date. I recommend incorporating the following reference, which is more relevant to the area of research:

Gonzales GF, Aguilar J, Yucra S, Vásquez-Velásquez C. A novel diagnostic approach to differentiate iron overload from inflammation in children using transferrin saturation (TSAT) and ferritin-based indices: A cross-sectional study. Sci Prog. 2025 Oct-Dec;108(4):368504251385071. doi: 10.1177/00368504251385071.

- A reference should be provided to support the statements in lines 79–82.

Methodology

- The study design is not clearly described. Although the authors indicate that the analysis is based on secondary data, this does not necessarily classify the study as retrospective.
- Definitions should be accompanied by appropriate references.
- In the statistical analysis section, please specify the measure of dispersion reported alongside the median.
- Evaluation of diagnostic validity would benefit from the use of logistic regression modeling and receiver operating characteristic (ROC) curve analysis. Alternatively, the bias-corrected Kappa index may be considered.
- The section on ethical considerations should be placed after the analysis plan, and should first emphasize the study design.
