# Supplementary material for: Characteristics of transferrin saturation and anemia-related biomarkers in patients with uterine adenomyosis
Source: PLoS One. 2026 Mar 17;21(3):e0344781. doi: 10.1371/journal.pone.0344781 (PMC12994800; doi:10.1371/journal.pone.0344781)
Supplement: Attachment — Submitted filename: 20260203_Response_to_Reviewers.docx [file pone.0344781.s005.docx]

**Response to Reviewers**

Title page

- Authors are advised to review the journal’s author guidelines and follow a classic structure when submitting manuscripts.

**Response:** Thank you for this suggestion. We reviewed the author guidelines of *PLOS ONE* and revised the manuscript layout to follow a standard structure.

Abstract

- A structured abstract is recommended.

**Response:** Following your recommendation, we agree have structured the abstract using the following subheadings: Background, Methods, Results, and Conclusions.

- Establish cut-off points for anemia and iron deficiency for Hb, Ferritin, TSAT.

**Response:** Thank you for this comment. We have now explicitly stated all prespecified cut-off values in the structured Abstract and the Definitions subsection of Methods. We defined anemia as Hb <11 g/dL to focus on clinically significant (moderate-to-severe) anemia, consistent with the WHO severity classification for non-pregnant women and the commonly used criteria in Japanese gynecologic practice.

- The abstract refers to a strong correlation; however, the corresponding quantitative values and p-values are not reported.

**Response:** We agree with your observation. We have added the relevant correlation coefficients and p-values in the Results subsection of the structured abstract to support the stated interpretation.

Introduction

- Lines 76 to 78 contain references that are not up to date. I recommend incorporating the following reference, which is more relevant to the area of research:

Gonzales GF, Aguilar J, Yucra S, Vásquez-Velásquez C. A novel diagnostic approach to differentiate iron overload from inflammation in children using transferrin saturation (TSAT) and ferritin-based indices: A cross-sectional study. Sci Prog. 2025 Oct-Dec;108(4):368504251385071. doi: 10.1177/00368504251385071.

**Response:** We appreciate this suggestion. We revised the statement on ferritin cut-offs in the Introduction and updated the supporting references by prioritizing recent guideline sources (e.g., WHO 2020; BSG 2021). Additionally, we incorporated the recommended study by Gonzales et al. (2025), discussing TSAT- and ferritin-based interpretation of iron status in the context of inflammation.

- A reference should be provided to support the statements in lines 79–82.

**Response:** Thank you for this helpful comment. We agree and have added appropriate references to support the description of TSAT as an indicator of iron availability (including the <20% threshold) and its calculation based on serum iron and total iron-binding capacity (TIBC).

Methodology

- The study design is not clearly described. Although the authors indicate that the analysis is based on secondary data, this does not necessarily classify the study as retrospective.

**Response:** Thank you for your suggestion. We clarified the study design by explicitly describing this work as a retrospective, cross-sectional, observational study based on electronic medical records from the first outpatient visit.

- Definitions should be accompanied by appropriate references.

**Response:** We appreciate this helpful comment. We added appropriate references to the Definitions subsection to support each operational definition and cut-off (Hb, ferritin, TSAT, and functional/overall iron deficiency).

- In the statistical analysis section, please specify the measure of dispersion reported alongside the median.

**Response:** Thank you for your suggestion. We clarified that continuous variables were summarized as medians (range) and categorical variables as n (%) in the Statistical analysis subsection.

- Evaluation of diagnostic validity would benefit from the use of logistic regression modeling and receiver operating characteristic (ROC) curve analysis. Alternatively, the bias-corrected Kappa index may be considered.

**Response:** Thank you for your thoughtful suggestion. Among patients with ferritin measured at the first visit (n=124), agreement between ferritin <20 ng/mL and TSAT <20% was fair (Cohen’s κ=0.375), with similar PABAK values (0.371). We performed multivariable logistic regression for TSAT-only classification (TSAT <20% with ferritin ≥20 ng/mL), adjusting for age and hypermenorrhea. Adenomyosis showed a trend toward higher odds compared with no uterine structural abnormality (OR 3.123, 95% CI 0.942–11.30; p=0.0688), whereas myoma was not associated (OR 1.234, 95% CI 0.399–4.079; p=0.7184). Model discrimination was modest (AUC=0.678, 95% CI 0.564–0.792; ROC in S1 Fig). Relevant text has been added to Methods and Results.

- The section on ethical considerations should be placed after the analysis plan, and should first emphasize the study design.

**Response:** We agree with your thoughtful comment. We reorganized the Methods section, placing the study design and statistical analysis before ethical considerations. Specifically, we placed the “Ethical approval” subsection after the “Statistical analysis” subsection.
