# Supplementary material for: Characteristics of transferrin saturation and anemia-related biomarkers in patients with uterine adenomyosis
Source: PLoS One. 2026 Mar 17;21(3):e0344781. doi: 10.1371/journal.pone.0344781 (PMC12994800; doi:10.1371/journal.pone.0344781)
Supplement: Attachment — Submitted filename: 20260215_response to editor.docx [file pone.0344781.s006.docx]

The reviewer has provided several comments; however, only one point requires your attention at this stage. According to the journal’s guidelines, the incorporation of coded data into an appropriate public repository is indicated. Please revise the Data Availability Statement accordingly and ensure compliance with the PLOS ONE data policy.

No other substantive revisions are required. We look forward to receiving your revised manuscript.

Response:

Thank you for giving us the opportunity to revise our manuscript.

We have uploaded our raw dataset to a public repository and revised the Data Availability Statement in the manuscript.
